# Migration-promoting role of VEGF-C and VEGF-C binding receptors in human breast cancer cells

**DOI:** 10.1038/sj.bjc.6603993

**Published:** 2007-10-02

**Authors:** A V Timoshenko, S Rastogi, P K Lala

**Affiliations:** 1 Departments of Anatomy and Cell Biology, The University of Western Ontario, London, Ontario N6A5C1, Canada; 2 Department of Biology, The University of Western Ontario, London, Ontario N6A5B7, Canada

**Keywords:** breast cancer, migration, VEGF-C, neuropilins, integrins

## Abstract

Vascular endothelial growth factor C (VEGF-C) is a lymphangiogenic factor over-expressed in highly metastatic, cyclooxygenase (COX)-2 expressing breast cancer cells. We tested the hypothesis that tumour-derived VEGF-C may play an autocrine role in metastasis by promoting cellular motility through one or more VEGF-C-binding receptors VEGFR-2, VEGFR-3, neuropilin (NRP)-1, NRP-2, and integrin *α*9*β*1. We investigated the expression of these receptors in several breast cancer cell lines (MDA-MB-231, Hs578T, SK-BR-3, T-47D, and MCF7) and their possible requirement in migration of two VEGF-C-secreting, highly metastatic lines MDA-MB-231 and Hs578T. While cell lines varied significantly in their expression of above VEGF-C receptors, migratory activity of MDA-MB-231 and Hs578T cells was linked to one or more of these receptors. Depletion of endogenous VEGF-C by treatments with a neutralising antibody, VEGF-C siRNA or inhibitors of Src, EGFR/Her2/neu and p38 MAP kinases which inhibited VEGF-C production, inhibited cellular migration, indicating the requirement of VEGF-C for migratory function. Migration was differentially attenuated by blocking or downregulation of different VEGF-C receptors, for example treatment with a VEGFR-2 tyrosine kinase inhibitor, NRP-1 and NRP-2 siRNA or *α*9*β*1 integrin antibody, indicating the participation of one or more of the receptors in cell motility. This novel role of tumour-derived VEGF-C indicates that breast cancer metastasis can be promoted by coordinated stimulation of lymphangiogenesis and enhanced migratory activity of breast cancer cells.

Vascular endothelial growth factor (VEGF)-C is the major lymphangiogenic factor which is expressed in certain normal tissues, for example large intestinal and mammary duct epithelia, skeletal and cardiac muscle, thyroid, ovary, and prostate (Joory *et al*, 2006) as well as in a variety of cancerous tissues including breast cancer (Kinoshita *et al*, 2001; Nakamura *et al*, 2003, 2005, 2006). Over-expression of VEGF-C in the tumour micro-environment has been reported to be associated with a poor prognosis (Nakamura *et al*, 2003; Mylona *et al*, 2007) and lymph node metastasis (Nakamura *et al*, 2005) in breast cancer patients. Although tumour-associated macrophages were reported to be a major source of VEGF-C in breast cancer (Schoppmann *et al*, 2006), we have recently discovered that cyclooxygenase (COX)-2 expressing, highly metastatic human breast cancer cells themselves secrete a copious amount of this factor in cell culture medium (Timoshenko *et al*, 2006). Tumour-derived VEGF-C is thought to promote tumour progression by inducing lymphangiogenesis and thereby lymph node metastasis (Saharinen *et al*, 2004; Timoshenko *et al*, 2006; Tobler and Detmar, 2006). As a lymphangiogenic factor, VEGF-C acts through activation of the tyrosine kinase receptor VEGFR-3 expressed by lymphatic endothelial cells (Saharinen *et al*, 2004). VEGF-C, however, can also bind to several other important cell membrane receptors such as VEGFR-2 (a major angiogenic receptor) (Joukov *et al*, 1996), neuropilin (NRP)-1 and NRP-2 (receptors for semaphorins) (Kärpänen *et al*, 2006), and *α*9*β*1 integrin (Vlahakis *et al*, 2005), a receptor for osteopontin, tenascin-C, and VCAM-1 (Marcinkiewicz *et al*, 2000). Expression of all these receptors, although typically noticed on endothelial cells, has also been reported in other cell types including tumour cells (Fitzpatrick *et al*, 2003; Vantyghem *et al*, 2005; Favier *et al*, 2006). These observations suggest that in addition to the major lymphangiogenic function, VEGF-C may play a role as an autocrine molecule directly affecting functions of certain cancer cells which express any of these VEGF-C-binding receptors.

Migration of cancer cells is an essential step for invasion and metastasis and can be promoted in an autocrine manner by various endogenous factors including VEGF-A (Dias *et al*, 2000; Bachelder *et al*, 2002). Recently, exogenous VEGF-C was shown to promote migration of Kaposi's sarcoma cells which expressed VEGFR-2 and VEGFR-3 (Marchio *et al*, 1999). NRP-2 can contribute to cell migration in collaboration with VEGFR-2 and VEGFR-3; NRP-2 ligand semaphorin-3F and NRP siRNA both inhibited the response of human microvascular endothelial cells (HMVEC) to VEGF-A and VEGF-C (Favier *et al*, 2006). The role of NRP-1 in migration was also demonstrated for a human colon adenocarcinoma cell line WiDR, which showed a significant drop in migratory activity after transfection with NRP-1 siRNA (Ochiumi *et al*, 2006). The principal demonstrated function of *α*9*β*1 integrin is acceleration of leucocyte migration, an effect that depends on unique sequences within the *α*9 cytoplasmic domain (Shang *et al*, 1999; Young *et al*, 2001; Chen *et al*, 2004). This integrin is over-expressed in an aggressive human breast cancer cell line 468LN capable of producing lymph node metastasis in nude mice (Vantyghem *et al*, 2005), suggestive of participation of VEGF-C. It has remained, however, unknown whether and how VEGF-C-binding receptors participate in mediating migration of VEGF-C-producing breast cancer cells.

We have shown that COX-2-mediated VEGF-C upregulation in human breast cancer served as a stimulus for lymphangiogenesis, a vehicle for lymphatic metastasis (Timoshenko *et al*, 2006). In that study, we found that high COX-2 expressing human breast cancer cells produced much higher levels of VEGF-C than VEGF-A. Present study was designed to explore a possible autocrine role of VEGF-C in breast cancer cell migration, including a systematic analysis of expression and migration-associated function of VEGF-C-binding receptors in a number of human breast cancer cell lines.

## MATERIALS AND METHODS

### Reagents

PP1 (Src kinase inhibitor) was purchased from Biomol (Plymouth Meeting, PA, USA). SU5416 (VEGFR-2 tyrosine kinase inhibitor), and Sigma FAST 3,3′-diaminobenzidine tablet sets were from Sigma (Oakville, ON, Canada). PD153035 (EGFR and Her2/neu tyrosine kinase inhibitor) and SB203580 (p38 kinase inhibitor) were from Calbiochem (San Diego, CA, USA). NRP-1 and NRP-2 siRNA duplexes, goat polyclonal anti-human VEGF-C antibody (sc-1881), and normal goat immunoglobulin G (IgG) (sc-2028) were from Santa Cruz Biotechnology (Santa Cruz, CA, USA). Rabbit polyclonal anti-human VEGFR-2 antibody (cat. no. 2479) was from Cell Signaling (Danvers, MA, USA). DMEM, *α*MEM, fetal bovine serum (FBS), Ca^2+^, Mg^2+^-free Dulbecco's phosphate-buffered saline (DPBS), TRIzol reagent and SuperScript II reverse transcriptase were from Invitrogen (Burlington, ON, USA). VEGF-C siGENOME SMARTpool of four siRNA duplexes, siCONTROL non-targeted siRNA, and DharmaFECT 2 transfection reagent were from Dharmacon (Lafayette, CO, USA). Mouse anti-integrin *α*9*β*1 monoclonal antibody (clones Y9A2, cat. no. MAB2078Z), goat anti-mouse IgG conjugated to R-phycoerythrin (cat. no. AQ196H), and negative isotype control for flow cytometry mouse IgG1 (cat. no. CBL600) were from Chemicon International (Temecula, CA, USA).

### Human breast cancer cell lines

The original source of all human breast cancer cell lines (MDA-MB-231, Hs578T, MCF7, T-47D, and SK-BR-3) was the ATCC (Manassas, VA, USA) excepting 468LN which was kindly provided by Dr Ann Chambers (London Regional Cancer Program, London, ON, Canada). This cell line was derived by Dr Chambers' group by *in vivo* selection of MDA-MB-468 cancer cell line for lymphatic metastasis (Vantyghem *et al*, 2005). All cell lines were maintained in DMEM supplemented with 10% FBS, 25 mM HEPES buffer, 50 U ml^−1^ penicillin, and 50 *μ*g ml^−1^ streptomycin except for 468LN, in which case DMEM was replaced with *α*MEM, and SK-BR-3, in which case DMEM was replaced with McCoy's 5A medium (modified).

### RT–PCR

Total RNA was extracted from breast cancer cells grown in 6-well plates by TRIzol reagent and cDNAs were synthesised using SuperScript II Reverse Transcriptase as described elsewhere (Timoshenko *et al*, 2006). Primers for VEGFR-2, VEGFR-3, NRP-1, NRP-2, VEGF-C, and GAPDH (Table 1) were synthesised locally at the UWO Oligo Factory (London, ON, Canada) and their quality was verified by a conventional PCR using GeneAmp PCR System from Perkin Elmer (Norwalk, CT, USA) based on standard amplification conditions: 30–35 cycles of denaturation at 94°C (30 s), annealing at 55°C (30 s), extension at 72°C (45 s) followed by 5 min of final extension at 72°C. Real-time quantitative PCR (qPCR) was performed in single microcapillary tubes using the LightCycler (Roche Diagnostic, Laval, Que., Canada) and SYBR Green Tag ReadyMix (Sigma, Oakville, ON, Canada) as previously described (Timoshenko *et al*, 2006). All data were normalised relative to the expression of GAPDH mRNA in respective samples.

### siRNA transfection

All siRNA transfection experiments were performed in antibiotic-free medium with cells grown in 6-well plates. The cells (1.5 × 10^5^ cells per well) were plated overnight at 37°C, 5% CO_2_ and then transfected with 100 nM of either siControl non-targeting siRNA or target-specific siRNAs to knock-down VEGF-C, NRP-1, or NRP-2 in the presence of 0.2% of DharmaFECT 2. The efficiency of transfection was assayed by qPCR or conventional PCR and, in addition, by ELISA for VEGF-C protein secretion.

### Flow cytometry

Flow cytometry analysis was performed based on the staining procedure described elsewhere (Vantyghem *et al*, 2005). Cells were grown up to 80% confluency in T75 flasks, gently dislodged with Trypsin-EDTA solution (0.5% trypsin, 0.48 mM EDTA·4Na in DPBS), centrifuged and resuspended in 2% FBS/DPBS flow buffer. All following steps were performed at 4°C or on ice. Aliquots of unfixed cells in flow buffer (100 *μ*l, 10^6^ cells) in Eppendorf tubes were incubated subsequently with mouse anti-*α*9*β*1 integrin primary antibody or isotype-matched control mouse IgG1 (1 *μ*g per 10^6^ cells) and secondary antibody labelled with R-phycoerythrin (0.5 *μ*g per 10^6^ cells) for 1 h every time on a rotating plate in a dark. Following every treatment, the cells were washed twice in 1 ml of flow buffer (4 min, 230 **g**) on a centrifuge Allegra X-22R from Beckman Coulter (Mississauga, ON, Canada). Finally, the stained cells (1 ml in flow buffer) were filtered through 40 *μ*m nylon cell strainers from BD Biosciences (Bedford, MA, USA) and analysed on a FACSCalibur cytometer (Becton Dickinson, San Jose, CA, USA) at the London Regional Flow Cytometry Facility (Robarts Research Institute, London, ON, Canada).

### Migration (chemokinesis) assay

The migration of MDA-MB-231 and Hs578T cells through polycarbonate membranes (having 8 *μ*m diameter pores) in 24 well Transwell cell culture chambers (Corning Costar Corporation, Cambridge, MA, USA) was quantified as described elsewhere (Timoshenko *et al*, 2003) with minor modifications. Briefly, 20 000 cells in an antibiotic-free DMEM supplemented with 0.1% FBS and respective treatments were placed in the upper insert and allowed to migrate for 24 h at 37°C in a humidified CO_2_ incubator; the bottom well was filled with the same solution used to resuspend cells. Cells that migrated and adhered to the bottom surface of the membranes were fixed with methanol (2 min) and stained for 5 min each with eosin and thiazine using Hemacolor kit from EM Science (Gibbstown, NJ, USA). The stained membranes were cut out, placed on a glass slide, and the number of migrant cells on the bottom surface of the membrane was counted using a bright field light microscope. The migrant cell number in the experimental (treated) wells expressed as a percentage of the control (untreated) wells provided the migration index. Each treatment was performed in triplicate or quadruplicate.

### Immunostaining for VEGFR-2

MDA-MB-231 cells were grown up to subconfluency on Lab-Tek Permanox slides with four chambers from Nalge Nunc (Naperville, IL, USA). The cell monolayers were rinsed with DPBS, fixed in 2% formaldehyde for 15 min, rinsed again 3 times with DPBS, and blocked with diluted goat normal serum for 1 h at room temperature. Primary polyclonal rabbit anti-VEGFR-2 antibody was diluted 1 : 100 in DPBS containing 0.3% triton X-100 and added to every chamber (500 *μ*l per well) for overnight incubation at 4°C. Then the cells were rinsed 3 times with DPBS and the VECTASTAIN Elite kit from Vector Laboratories (Burlingame, CA, USA) was applied to stain samples according the manufacturers' protocol. To visualise the sites of immunostaining, 3,3′-diaminobenzidine/urea hydrogen peroxide substrate solution was used and, finally, the samples were counterstained with Harris' alum hematoxylin (EMD Chemicals, Darmstadt, Germany) and mounted with Faramount (DakoCytomation, Glostrup, Denmark).

### Statistics

Data were analysed by two way ANOVA and Student's *T*-test considering *P*<0.05 as an indicator of significant difference between means.

## RESULTS

### VEGF-C function blocking antibody, VEGF-C siRNA, and VEGF-C synthesis inhibitors inhibit migration of human breast cancer cells

As we have recently reported, highly metastatic MDA-MB-231 and Hs578T cells secrete a relatively high amount of VEGF-C in cell culture medium (Timoshenko *et al*, 2006). To find out whether migration of these human breast cancer cell lines depends on endogenously produced VEGF-C, we used three different approaches, namely (1) the treatment of cells with a VEGF-C neutralising/function blocking antibody, (2) transfection of cells with VEGF-C siRNA to downregulate gene expression, and (3) the treatment of cells with kinase inhibitors for EGFR/Her2/neu PD153035), Src (PPI), and p38 MAPK (SB203580) at non-toxic concentrations which have recently been shown to inhibit VEGF-C production by MDA-MB-231 cells (Timoshenko *et al*, 2006).

**Figure 1:**
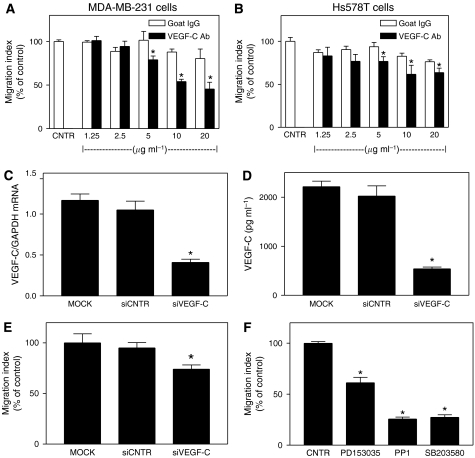
(**A** and **B**) Effects of different concentrations of a polyclonal, function blocking antibody against human Vascular endothelial growth factor (VEGF)-C on migration of VEGF-C-secreting MDA-MB-231 and Hs578T cells cultured for 24 h in serum-free DMEM supplemented with 0.1% FBS. A strong inhibition of migration (*P*<0.02), noted at all antibody concentrations ranging between 5 and 20 *μ*g ml^−1^, and no inhibition with similar concentrations of control goat immunoglobulin G (IgG), indicated an autocrine migration-promoting role of VEGF-C in these cells. (**C**) Effects of siVEGF-C (100 nM) treatments on the expression of VEGF-C gene in MDA-MB-231 cells (48 h). (**D**) VEGF-C secretion from MDA-MB-231 cells transfected with siVEGF-C (100 nM, 48 h) which were incubated in serum-free DMEM for other 24 h. (**E**) Migration of MDA-MB-231 cells is inhibited by transfection with siVEGF-C (100 nM) as measured at 24 h time point in DMEM supplemented with 0.1% FBS. (**F**) Effects of kinase inhibitors for EGFR/Her2/Neu (PD153035), Src (PP1), and p38 MAPK (SB203580) on migration of MDA-MB-231 cells cultured for 24 h in DMEM supplemented with 0.1% FBS. Data represent mean±s.d. (*n*=4). ^*^P<0.01. All the above kinase inhibitors at the tested non-toxic concentrations had no significant effect on cell proliferation/survival and inhibited VEGF-C production, as we reported earlier (Timoshenko *et al*, 2006).

First of all, we screened several commercially available anti-human VEGF-C antibodies for function blocking activity and found that a polyclonal goat antibody raised against the C terminus of VEGF-C of human origin (Santa Cruz, CA, USA) was active, similar to its blocking activity reported on coronary endothelial tube formation from embryonic cardiac explants (Tomanek *et al*, 2002). In view of the fact that this antibody recognises the VEGF-C propeptide as well as several cleaved products inclusive of the C terminus (Siegfried *et al*, 2003; Tang *et al*, 2003), the VEGF-C function blocking activity of this antibody is possibly attributed to a protection of the antigen–antibody complex from further proteolytic cleavage that would generate VEGF-C peptides capable of activating VEGF-C receptors. Using this antibody, we treated COX-2/VEGF-C-expressing MDA-MB-231 and Hs578T cells at different concentrations (0–20 *μ*g ml^−1^) and allowed them to migrate for 24 h at 37°C. A significant (*P*<0.001) decrease in migration resulted from the antibody treatment of both cell lines at concentrations of 5 *μ*g ml^−1^ and higher, whereas control goat IgG at similar concentrations had no effect (Figures 1A and B). In contrast, at similar antibody concentrations, no significant effect was noted on cell proliferation/survival (MTT assay) at 24 h (not shown). Thus, secreted VEGF-C is an important factor maintaining the migratory (but not proliferative) function of these breast cancer cell lines.

Secondly, to silence VEGF-C gene expression, MDA-MB-231 cells were transfected with VEGF-C siRNA (100 nM) and the following three parameters were examined: VEGF-C mRNA expression (Figure 1C), VEGF-C proteins secretion (Figure 1D), and cell migration (Figure 1E). In all cases, we observed a significant drop of every parameter when the specific siRNA-transfected cells were compared with either mock-transfected control or scrambled siRNA treated cells. The inhibition of cell migration, although significant, was rather moderate in comparison with the effect of neutralising antibody, evidently due to the fact that VEGF-C production could not be completely knocked-down. Nevertheless, the migration-inhibitory effects of VEGF-C siRNA on VEGF-C producing MDA-MB-231 cells strongly supports the migration-promoting role endogenous VEGF-C.

Finally, we treated MDA-MB-231 cells with PD153035 (EGFR and Her2/neu tyrosine kinase inhibitor), PP1 (Src kinase inhibitor), and SB203580 (p38 MAP kinase inhibitor) which had earlier been shown to significantly inhibit VEGF-C secretion by this cell line at the respective concentrations without any effect on cell proliferation/survival (Timoshenko *et al*, 2006). As shown in Figure 1F, all these inhibitors at non-toxic concentrations known to inhibit VEGF-C secretion (Timoshenko *et al*, 2006) also inhibited the migration of MDA-MB-231 cells in a parallel manner at a 24 h time point. The data suggest that these kinases are important for both the cellular events (VEGF-C secretion and cell migration) or for the event of VEGF-C secretion, which in turn, regulated cellular migration. While on their own, these data do not prove the autocrine role of VEGF-C in cellular migration, they are supportive of earlier experiments using the function blocking antibody and VEGF-C siRNA.

Since VEGF-C can act through different VEGF-C-binding receptors, the next issue was to determine the expression and the role of specific receptors in mediating migratory responses in breast cancer cells.

### Expression of VEGF-C-binding receptors in human breast cancer cell lines

The biological action of VEGF-C can be mediated theoretically through two tyrosine kinase receptors VEGFR-2 and VEGFR-3 (Joukov *et al*, 1996), NRP-1 and-2 (Kärpänen *et al*, 2006), and *α*9*β*1 integrin (Vlahakis *et al*, 2005). To find out, which of these receptors may be instrumental for autocrine effects of VEGF-C, we analysed their mRNA or protein expression in five human breast cancer cell lines (MCF-7, T-47D, SK-BR-3, Hs578T, and MDA-MB-231), which differ in their expression of COX-2 and VEGF-C (Timoshenko *et al*, 2006).

**Figure 2:**
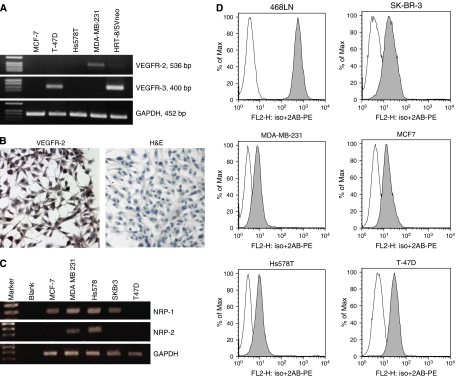
(**A**) Expression of VEGFR-2 and VEGFR-3 mRNA in human breast cancer cells (MCF-7, T-47D, Hs578T, and MDA-MB-231) and a human trophoblast cell line (HTR-8/SVneo, used as a positive control for VEGFR-3) as revealed by RT–PCR. MDA-MB-231 cells express VEGFR-2 (binding to VEGF-A and -C) but not VEGFR-3 (binding to VEGF-C and -D). MCF-7 and Hs578T cells expressed none of these receptors, whereas T-47D expressed VEGFR-3. (**B**) Immunostaining of MDA-MB-231 cells for VEGFR-2 (image on the left) and its control (image on the right), cells counterstained with hematoxylin and eosin (H&E). (**C**) Expression of neuropilin (NRP)-1 and NRP-2 mRNAs in human breast cancer cell lines MCF-7, MDA-MB-231, Hs578T, SK-BR-3, and T-47D as revealed by RT–PCR. (**D**) Flow cytometry analysis of various human breast cancer cells (468LN, MDA-MB-231, Hs578T, SK-BR-3, MCF7, and T-47D) labelled with *α*9*β*1 integrin antibody. In comparison with strongly *α*9*β*1 integrin positive 468LN cells (15), other cell lines demonstrated relatively low levels of *α*9*β*1 integrin expression (filled profiles) overlapping partially with non-specific isotype control staining (opened profiles). Approximately 20 000 cells were analysed in each case.

Non-metastatic, COX-2-negative, poorly migratory and low VEGF-C secreting MCF-7 cells as well as moderately COX-2 expressing and VEGF-C secreting Hs578T cells expressed neither detectable VEGFR-2 nor VEGFR-3 mRNA (Figure 2A). Low COX-2 expressing and VEGF-C secreting T-47D cells expressed VEGFR-3 but not VEGFR-2 mRNA, whereas high COX-2 expressing and VEGF-C secreting, highly migratory/invasive and metastatic MDA-MB-231 cells expressed VEGFR-2 but not VEGFR-3 mRNAs (Figure 2A). Thus, the expression of VEGFR-2 and VEGFR-3 bore no relationship to the level of COX-2 expression or VEGF-C secretory ability of breast cancer cell lines. The VEGFR-2 protein expression was confirmed in highly metastatic MDA-MB-231 cells by immunocytochemistry (Figure 2B).

With regard to NRPs, the expression of NRP-1 mRNA was found to be ubiquitous in all the tested human breast cancer cell lines, whereas NPR-2 mRNA was expressed only by high COX-2 and VEGF-C expressing MDA-MB-231 and Hs578T cells (Figure 2C).

To screen the expression of *α*9*β*1 integrin, we used flow cytometry analysis of several human breast cancer cell lines, inclusive of a high *α*9*β*1 integrin-expressing 468LN cell line (Vantyghem *et al*, 2005), used as a positive control. Figure 2D shows that all the tested human breast cancer cell lines (MDA-MB-231, Hs578T, SK-BR-3, T-47D, and MCF7) expressed relatively low levels of *α*9*β*1 integrin in comparison with 468LN. Thus, the ratio of geometric means of fluorescence intensity for cell population treated with anti-*α*9*β*1 integrin primary antibody to those treated with isotype-matched normal mouse IgG1 were 170 for 468LN, 5.5 for T-47D, 4.7 for SK-BR-3, 3.5 for MCF7, 3.1 for Hs578T, and 2.7 for MDA-MB-231.

Thus, human breast cancer cell lines are heterogeneous in their expression of different VEGF-C-binding receptors, which can potentially mediate autocrine action of VEGF-C including its ability to stimulate cell migration, as documented in other cell types, for example endothelial cells (Makinen *et al*, 2001), lung cancer cells (Tanno *et al*, 2004), Kaposi's sarcoma cells (Marchio *et al*, 1999), and leucocytes (Shang *et al*, 1999; Young *et al*, 2001; Chen *et al*, 2004).

### VEGFR-2 contributes to migration of MDA-MB-231 cells

**Figure 3:**
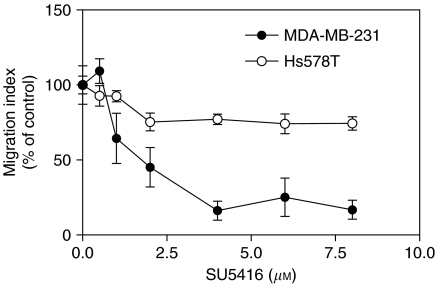
Effect of SU5416, a selective inhibitor of VEGFR-2 (Flk-1/KDR) tyrosine kinase, on migration of MDA-MB-231 and Hs578T cells cultured for 24 h in DMEM supplemented with 0.1% FBS. Data represent mean±s.d. (*n*=3). ^*^P<0.02. The migration of VEGFR-2 expressing MDA-MB-231 cells was strongly inhibited at inhibitor concentrations ⩾2 *μ*M, while only a very minor effect was noted with VEGFR-2-negative Hs578T cells.

Since MDA-MB-231 cells secreted the highest level of VEGF-C (Timoshenko *et al*, 2006) and expressed both VEGFR-2 mRNA and protein (Figure 2A and B), we tested the role of this receptor in cell migration using a selective pharmacological inhibitor of VEGFR-2 tyrosine kinase SU5416 (Fong *et al*, 1999). Treating cells with SU 5416 resulted in a concentration-dependent inhibition of cellular migration without any significant effect on cell proliferation/survival, indicating the requirement of VEGFR-2 in migratory function of these cells (Figure 3). In particular, at a concentration of 4 *μ*M and higher SU5416 inhibited migration of MDA-MB-231 cells at 24 h by approximately 80% (*P*<0.001). The specificity of SU5416 was further validated by the fact that VEGFR-2 negative Hs578T cells showed very minor inhibitory response to SU5416 (Figure 3). These results affirm the role of one or more of the autocrine ligands (VEGF-A, VEGF-C) interacting with VEGFR-2 in migration promotion of MDA-MB-231 cells. It is also possible that receptor(s) other than VEGFR-2 may also be involved. With this point in mind, we analysed the role of NRP-1 and NRP-2 which are expressed in MDA-MB-231 and HS578T cells.

### Contribution of NRP-1 and NRP-2 to migration of human breast cancer cells

**Figure 4:**
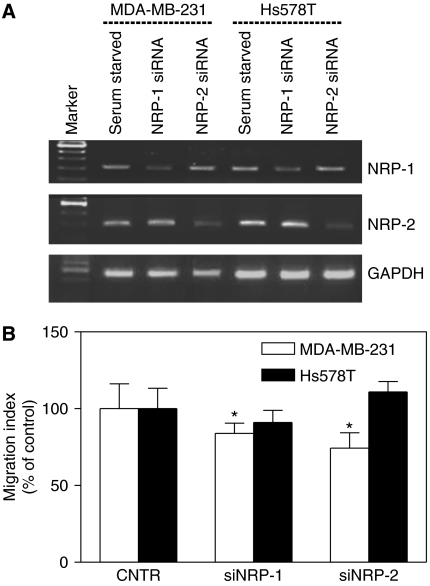
(**A**) Silencing of neuropilin (NRP)-1 and neuropilin (NRP)-2 mRNA expression in MDA-MB-231 and Hs578T cells transfected with 100 nM of respective siRNAs for 48 h (see Material and Methods). The RT–PCR data demonstrated a highly specific knock-down of both genes. (**B**) Migration of untreated (mock-transfected control) and NRP-1 and NRP-2 siRNA-transfected MDA-MB-231 and Hs578T cells. Whereas both siRNAs inhibited (^*^P<0.05) migration of VEGFR-2-expressing MDA-MB-231 cells, the migratory activity of transfected Hs578T cells was not impaired.

To evaluate the contribution of these receptors in the migration of VEGF-C-secreting MDA-MB-231 and Hs578T cells, we measured the migratory activity of these cells after transfection with respective siRNAs. As shown in Figure 4A, siNRP-1 and siNRP-2 specifically silenced the expression of NRP-1 and NRP-2 mRNAs in both cell lines. The migration of VEGFR-2 expressing MDA-MB-231 cells was inhibited moderately after siRNA-mediated knock-down of either NRP-1 or NRP-2 genes whereas no migration-inhibitory effect of siRNA treatment was noted in the case of VEGFR-2-negative Hs578T cells (Figure 4B).

### Effect of *α*9*β*1 integrin antibody on migration of human breast cancer cells

**Figure 5:**
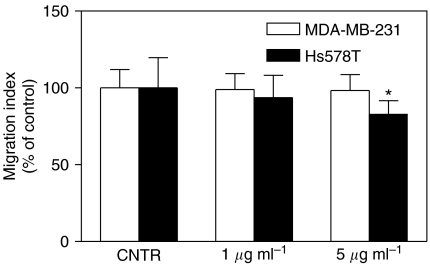
Effects of *α*9*β*1 integrin function-blocking antibody on migration of MDA-MB-231 and Hs578T breast cancer cells at a 24 h. The antibody (1 and 5 *μ*g ml^−1^) caused a concentration dependent inhibition of migration of Hs578T but not MDA-MB-231 cells. ^*^P<0.05.

To test the role of *α*9*β*1 integrin in migration of MDA-MB-231 and Hs578T cells (both of which expressed this integrin at low levels), we analysed their migration in the presence of two concentrations of mouse anti-integrin *α*9*β*1 monoclonal antibody (1 and 5 *μ*g ml^−1^). This antibody was reported to inhibit VEGF-C/-D-mediated migration of *α*9-transfected mouse embryonic cells and primary adult human dermal microvascular endothelial cells (Vlahakis *et al*, 2005). As noted in Figure 5, migration of Hs578T cells but not MDA-MB-231 cells was significantly inhibited in the presence of the antibody at a concentration of 5 *μ*g ml^−1^.

## DISCUSSION

Present study, to our knowledge, is the first one to demonstrate the autocrine role of VEGF-C in promoting human breast cancer cell migration, a critical step for invasion and metastasis. The migration-stimulating role of endogenous VEGF-C was demonstrated with two approaches: use of neutralising antibody and silencing of VEGF-C gene with siRNA. The third approach of inhibition of VEGF-C production by certain signalling inhibitors provided further support to these data. These findings are highly relevant for human breast cancer progression and metastasis because of two reasons: (a) we have shown that VEGF-C is the dominant product of highly metastatic human breast cancer cells amongst various members of the VEGF family, upregulated by COX-2 – an important marker for breast cancer progression (Timoshenko *et al*, 2006); (b) VEGF-C expression in human breast cancer is associated with poor prognosis (Nakamura *et al*, 2003; Mylona *et al*, 2007). Thus, VEGF-C plays a dual role in promoting breast cancer progression: a stimulation of lymphangiogenesis and thereby lymphatic metastasis (Saharinen *et al*, 2004; Timoshenko *et al*, 2006; Tobler and Detmar, 2006), and a direct action on cancer cells in stimulating cellular migratory function. Indeed, our recent studies of quantitative immunocytochemistry of VEGF-C protein expression in human breast cancer cells *in situ* revealed no significant difference of expression levels between lymph node positive and negative specimens, suggesting additional lymphangiogenesis-independent role(s) of VEGF-C (Lala *et al*, 2007). We have further shown in the present study that the autocrine migration stimulatory role of VEGF-C is mediated by multiple VEGF-C receptors expressed by breast cancer cells. It is highly likely that breast cancer cells *in situ* are also heterogeneous in expression of different VEGF-C receptors, similar to the breast cancer cell lines employed in the present study, and the receptor bearing cells would respond to both endogenous and exogenous (produced by other cells such as macrophages (Schoppmann *et al*, 2006) in the breast cancer stroma) VEGF-C in the tumour microenvironment.

Central physiological functions of VEGF family ligands and their receptors as crucial regulators of vasculogenesis, angiogenesis, lymphangiogenesis, and vascular permeability have been well documented during last decade (Roy *et al*, 2006; Shibuya and Claesson-Welsh, 2006). While VEGF-C has not been described before as an autocrine factor in promoting tumour progression, recent studies in a variety of tumours have reported angiogenesis-independent roles of VEGF-A, another important member of the VEGF family, in promoting tumour cell proliferation/survival and migration. For example, an autocrine function of VEGF-A has been demonstrated for the following: growth and migration of leukaemia cells (Dias *et al*, 2000), proliferation of Kaposi's sarcoma, melanoma, and ovarian carcinoma cell lines (Masood *et al*, 2001), proliferation of interleukin 6-treated prostate cancer cells (Steiner *et al*, 2004), growth of a human gastric adenocarcinoma cell line MGC803 (Tian *et al*, 2001), and of malignant pleural mesothelioma (Strizzi *et al*, 2001). Finally, VEGF-A has been show to promote survival, migration, and invasiveness of breast cancer cells (Mercurio *et al*, 2005). The receptor responsible for many of the VEGF-A actions cited above has been identified as VEGFR-2, which can also serve as a receptor for VEGF-C (McColl *et al*, 2004). The 21 kDa peptide derived after full processing of VEGF-C is the only VEGF-C peptide capable of activating VEGFR-2 (Joukov *et al*, 1997), and this peptide has been demonstrated in the conditioned medium of MDA-MB-231 cells (Nakamura *et al*, 2006) which we have shown to express VEGFR-2 but not VEGFR-3. In the present study, migration inhibition in the presence of the VEGFR-2 inhibitor may be due to the presence of either of the endogenous ligands, VEGF-A, or VEGF-C. While the roles of VEGF-A in tumour progression have received a lot of attention, less attention has been devoted to VEGF-C. In situations, where the VEGF-A/VEGF-C balance in the tumour microenvironment is shifted in favour of VEGF-C, the VEGF-C-mediated responses would likely dominate. For example, higher serum concentrations of VEGF-C than that of VEGF-A, believed to be tumour derived, correlates with lymph node metastasis in patients with squamous cell carcinoma of the oesophagus (Krzystek-Korpacka *et al*, 2007). The rate of secretion of VEGF-C *in vitro* by the highly metastatic human breast cancer cell line MDA-MB-231 was found to be 10 times than that of VEGF-A (Timoshenko *et al*, 2006). Whether this is true for COX-2 expressing breast cancer *in vivo* remains unknown at present.

While the traditional endothelial cell receptors for VEGF-C are VEGFR-3 and VEGFR-2, respectively, responsible for lymphangiogenic and angiogenic events, recently other VEGF-C binding receptors including NRP-1 and NRP-2 (Kärpänen *et al*, 2006) have also been shown to mediate VEGF-C action. We found a partial requirement of NRP-1 and NRP-2 for migratory function of MDA-MB-231 cells, but not Hs578T cells. The mechanisms of NRP action are complex. NRP-1 and NRP-2 are receptors for semaphorins, and can antagonise semaphorin action by binding to and sequestering VEGFs (Ellis, 2006; Guttmann-Raviv *et al*, 2006). NRP-binding semaphorins 3A, 3B, and 3F exhibit antitumour properties, some also described as migration inhibitory molecules (Bachelder *et al*, 2003; Nasarre *et al*, 2005). Thus, NRP-mediated pro-migratory action on MDA-MB-231 breast cancer cells is likely a consequence of endogenous VEGF-C binding. Since semaphorins are expressed by breast cancer cell lines including MDA-MB-231, or present in the breast cancer micro-environment *in situ* (Christensen *et al*, 2005), we suggest that our *in vitro* findings of NRP action are of *in vivo* relevance. Differential expression of semaphorins may be responsible for the differential effects of NRP-1/-2 gene knock-down on cellular migration noted in MDA-MB-231 and Hs578T cells in the present study. Another possible reason of this finding is the differential expression of VEGFR-2 in these two cell lines (Figure 2A), since the need for a cooperation between NRPs and VEGFR-2 has been reported for certain NRP actions in other cells. NRP-1 and NRP-2 are non-tyrosine kinase receptors which are believed to transmit intracellular signals in conjunction with co-receptor complexes involving plexin, VEGFR-1, or VEGFR-2 (Ellis, 2006; Guttmann-Raviv *et al*, 2006; Ochiumi *et al*, 2006). VEGFR-2 has been reported to interact with NRP-2 and promote the survival and migration of HMVECs (Favier *et al*, 2006) and its interaction with NRP-1 is needed for migration of human vascular smooth muscle cells (Liu *et al*, 2005). Above-mentioned possibilities for differential NRP actions in our breast cancer cell lines remain to be tested.

Integrin *α*9*β*1 is another VEGF-C binding receptor expressed by neutrophils (Shang *et al*, 1999), human epithelial, and muscle cells (Palmer *et al*, 1993; Basora *et al*, 1998), and certain tumour cells and tissues (Palmer *et al*, 1993; Basora *et al*, 1998; Vantyghem *et al*, 2005). This receptor has been reported to signal via the *α*9 chain to promote cell migration (Young *et al*, 2001). Our results reveal that VEGF-C producing Hs578T cells expressed integrin *α*9*β*1 at a level higher than MDA-MB-231 cells. This is the only identifiable receptor contributing to the migratory function of Hs578T cells, as no VEGFR-2/-3 mRNA expression was seen in these cells and NRP-1/-2 siRNAs failed to affect the migration of these cells. Since Hs578T cells do not express osteopontin (Sharp *et al*, 1999) but express tenascin-C (Dandachi *et al*, 2001) – the two other ligands for integrin *α*9*β*1, the role of *α*9*β*1 in migration of these cells may be attributed to endogenous VEGF-C or tenascin-C, or both. However, since the inhibitory activity of *α*9*β*1 integrin antibody was significant but rather moderate in comparison with VEGF-C function blocking antibody, other unidentified VEGF-C binding receptors may also be involved.

In summary, the present study shows that the migratory function, an essential step for tumour invasion and metastasis, is promoted in an autocrine fashion by endogenous VEGF-C produced by metastatic human breast cancer cell lines, utilising multiple VEGF-C receptors. Further studies are needed to establish a firm link between the promigratory roles of VEGF-C and these receptors as well as to identify precise signalling mechanisms responsible for the autocrine role of VEGF-C mediated by different VEGF-C receptors. Combined with our earlier reported data that COX-2 and certain EP receptors (EP1 and EP4) are responsible for promotion of cellular migration (Timoshenko *et al*, 2003) as well as VEGF-C upregulation in human breast cancer (Timoshenko *et al*, 2006), present results reinforce the place of COX-2 inhibitors and specific EP antagonists in breast cancer chemo-intervention.

## Figures and Tables

**Table 1:** Oligonucleotide primer pairs for RT–PCR

**Gene (accession #)**	**Forward primer sequence, 5′ → 3′ (positions)**	**Reverse primer sequence, 5′ → 3′ (positions)**	**Product size (bp)**
VEGF-C (NM_005429)	CGGGAGGTGTGTATAGATGTG (830–850)	ATTGGCTGGGGAAGAGTTTG (1412–1393)	583
VEGFR-2 (NM_002253)	CAATGGAGGGGAACTGAAGAC (2679–2699)	TCTGGCTACTGGTGATGCTGT (3214–3194)	536
VEGFR-3 (NM_002020)	GAGCAGCCATTCATCAACAAG (427–447)	GGTAGTCCCAGTCAAAGGTG (826–807)	400
NRP-1 (NM_003873)	GATACGAAGGTGAAGGAG (3019–3036)	TATAGTTCTCCAGGGCAG (3222–3205)	204
NRP-2 (NM_201279)	GCAGATGAATACGAGGTG (3213–3230)	GCAGCACTTTTGGTGGTT (3509–3492)	297
GAPDH (NM_002046)	ACCACAGTCCATGCCATCAC (628–647)	TCCACCACCCTGTTGCTGTA (1079–1060)	452

VEGF=Vascular endothelial growth factor; NRP=neuropilin.
